# Process evaluation of a complex intervention to optimize quality of prescribing in nursing homes (COME-ON study)

**DOI:** 10.1186/s13012-019-0945-8

**Published:** 2019-12-11

**Authors:** Pauline Anrys, Goedele Strauven, Sandrine Roussel, Marie Vande Ginste, Jan De Lepeleire, Veerle Foulon, Anne Spinewine

**Affiliations:** 1 Clinical Pharmacy Research Group, Louvain Drug Research Institute, Université catholique de Louvain, UCLouvain, Av E Mounier, 72 B1.72.02, 1200 Brussels, Belgium; 2 Department of Pharmaceutical and Pharmacological Sciences, KU Leuven, Leuven, Belgium; 3 Department Public Health and Primary Care, ACHG, KU Leuven, Leuven, Belgium; 4 Pharmacy department, Université catholique de Louvain, CHU UCL Namur, Yvoir, Belgium

**Keywords:** Potentially inappropriate prescribing, Process evaluation, Complex intervention, Mixed methods, Nursing homes

## Abstract

**Background:**

The COME-ON study was a cluster-controlled trial of a complex intervention that consisted of a blended training program, local interdisciplinary meetings, and interdisciplinary case conferences in Belgian nursing homes. The intervention was associated with significant improvements in the appropriateness of prescribing. The aims of this study were to describe the implementation of the intervention and to explore the experiences of participants, for the purpose of identifying factors associated with implementation and perceived impact and to draw lessons for future implementation.

**Methods:**

We performed a mixed-method process evaluation. Questionnaires and reports were used to collect quantitative data on implementation and experiences from the 24 NHs and participating healthcare professionals (coordinating physicians, general practitioners, pharmacists, and nurses) in the intervention group. Multidisciplinary focus groups focusing on factors associated with implementation and perceived impact were conducted in 11 NHs.

**Results:**

Overall, the rate of implementation and the satisfaction of participants were good, despite some variability between NHs and HCPs. Although perceived impact on nursing home residents varied, most participants perceived a positive impact for themselves. Factors associated with implementation and perceived impact were identified at different levels: intervention, healthcare professionals, organization, and external context. The interdisciplinary and face-to-face approaches were recognized as key elements for the success of the intervention, despite organizational constraints. The attitude of general practitioners was identified both as a barrier to and a facilitator for implementation and its success. The professional role and competency of the pharmacist influenced perceived impact. The pre-existing relationships between HCPs and the presence of a leader facilitated implementation and perceived impact. Remuneration was deemed necessary for the study and for future implementation.

**Conclusions:**

Overall, the intervention, and more specifically its interdisciplinary aspect, was well implemented and appreciated by HCPs. This probably contributed to the positive effect on the appropriateness of prescribing. Future implementation must take into account the various factors found to affect implementation and perceived impact, in order to maximize effect and sustainability.

Trial registration

Current Controlled Trials ISRCTN66138978; registered 18 November 2015, retrospectively registered, https://www.isrctn.com/ISRCTN66138978

## Background

For many years, concerns have been raised about the high prevalence of polypharmacy and potentially inappropriate prescribing (PIP) among nursing home residents (NHRs) [1–3]. The presence of PIP has been associated with adverse drug reactions [4, 5], increased healthcare utilization [6–10], and even death [6–8, 11]. Reviews of studies on improving the appropriateness of prescribing in the nursing home (NH) setting have produced mixed results [12–18]. Complex interventions, i.e., interventions with multiple interacting components, are likely to be required [12–15]. However, there is a need for further high-quality trials to identify which interventions are most effective in which context [12–18].

Process evaluation is an essential part of designing and testing complex interventions [19, 20]. Besides complexity related to multiple interacting components, complexity can also be due to the difficulty of implementation and the number of organizational levels targeted [21]. Process evaluation within a randomized controlled trial (RCT) of a complex intervention makes it possible to interpret findings and to inform policymakers planning future implementation [22]. Moreover, studies assessing interventions that were context-dependent have lacked process information on, e.g., the level of implementation or the influencing contextual factors; this has made interpretation in systematic reviews difficult [14, 19, 23]. The NH environment and context can vary from one country, region, or NH to another and this can substantially influence the development and effect of approaches to improving appropriateness of prescribing. Process evaluations of RCTs on appropriate prescribing in the NH setting have been scarce [24].

The COME-ON (Collaborative approach to Optimize MEdication use for Older people in Nursing homes) study was a cluster-controlled trial in 54 Belgian NHs to evaluate the impact of a complex intervention on the appropriateness of prescribing [25]. The core element of the intervention was a structured and repeated interdisciplinary review of residents’ medication (referred to hereafter as “interdisciplinary case conferences” (ICCs)) supported by a blended training program and local interdisciplinary meetings (LIMs; referred as to “local concertation” in the protocol paper). General practitioners (GPs), coordinating physicians, nurses, and pharmacists were involved in all components of the intervention. Details about each component of the intervention are provided in Table 1. The protocol has been published previously [26].

**Table 1:** COME-ON intervention components

Intervention component	Description	Objectives	Participants involved	Moment and frequency	Incentives
1. Blended training					
1a. Training—e-learning	Module 1: Pharmacotherapy in older people; potentially inappropriate medications and tools used to measure it Module 2: What is a medication review? How can HCP contribute? Module 3: How to perform an interdisciplinary medication review? Module 4: Teamwork	(a) to provide the key messages about the pharmacotherapy in older people (b) to explain how to conduct an interdisciplinary medication review + provide tools	Physicians Nurses Pharmacists	To be done preferentially over months 1 and 2, but available during whole study period	Accreditation for GPs and pharmacists; certificate of attendance for nurses
1b. Training—interdisciplinary workshops	Interdisciplinary workshops, provided by the research team. Problem-based learning using clinical vignettes: How to conduct an interdisciplinary medication review? What is the contribution of each HCP? How to classify DRPs?	(a) to explain how to conduct an interdisciplinary medication review + provide tools (b) to initiate teamwork and communication between HCPs of the same NH	Physicians Nurses Pharmacists	At month 2	Accreditation for GPs and pharmacists; certificate of attendance for nurses
	Specific training for pharmacists, provided by the research team: How to prepare a medication review? How to make a suggestion? Where to find relevant information about medications?	(a) to support pharmacists to take up their role in the interdisciplinary team	Pharmacists	At month 2	Accreditation for pharmacists
	Specific training for nurses, provided by the coordinating physician and/or pharmacist: ▪ Detection of adverse drug events by nurses ▪ Drug administration	(a) to strengthen the competencies of the nurses	Nurses	Twice or more during the study period at the discretion of the CP	Certificate of attendance
2. Local interdisciplinary meetings	Interdisciplinary discussion about the rational use of one specific class of medications at the level of the nursing home. Material was provided by the research team about the antidepressants and the lipid-lowering drugs (summary of evidence available + topics to be discussed). Possibility to invite external expert(s).	(a) to reach consensus on the appropriate use of one specific medication class within each NH and (b) to initiate teamwork and communication between HCPs of the same NH	Physicians Nurses Pharmacists	Two meetings of approximately 2 hours during the study	Remuneration for all HCPs involved in the COME-ON study
3. Interdisciplinary case conferences	Interdisciplinary face-to-face medication reviews of all medications for each included NHR	To perform a medication review of all medications taken by the resident and to plan / evaluate interventions to optimize medication use	Physicians Nurses Pharmacists	Once every 4 months (i.e., three time over study period) for a duration of approximately 20 minutes/ICC/NHR	Remuneration for all HCPs

The primary outcome measure related to the appropriateness of prescribing at resident level and was considered successful when at least one potentially inappropriate medication (PIM) or potential prescribing omission (PPO) present at baseline had been solved at the end of study and when there were no new PIMs or PPOs at the end of study compared with baseline. Explicit tools were used and incorporated in an algorithm to detect PIMs and PPOs. Using a three-level mixed-effects model accounting for data clustering, a significant effect in favor of the intervention was observed (odds ratio 1.479 [95%CI 1.062–2.059, *p* = 0.021]; number needed to treat 19). Analysis of PIMs at a criterion level showed slightly better results for the intervention group for some criteria (e.g., criteria related to the use of PPIs or benzodiazepines). Underuse of vitamin D substantially decreased in the intervention group [27].

With regard to medication use data, the median number of medications did not change over time in either group. However, significant decreases in the consumption of antithrombotic agents, drugs for peptic ulcer and gastroesophageal reflux disease, hypnotics, and sedatives were observed in the intervention group but not in the control group. The use of lipid-modifying agents decreased in both groups, but much more in the intervention group. In the intervention group, a significant increase was seen in the use of vitamin A/D. Most clinical outcomes measured (including hospital admission, visit to the emergency department, consultation with GP or specialist physician) did not significantly differ between groups. Data on clinical outcomes must however be interpreted with great caution for different reasons, and these are discussed in detail elsewhere [27].

The aim of the present paper is to describe the process evaluation of the COME-ON study. Specifically, the objectives were to understand how the intervention was implemented and to explore the experiences of participants, for the purpose of identifying factors associated with implementation and perceived impact and to draw lessons for future implementation.

## Methods

### Setting

Each Belgian NH is legally bound to have a coordinating physician—a GP with specific additional training—who is, among other things, responsible for promoting rational medication use within the NH through the development of a therapeutic formulary. NHRs have the right to choose their GPs and the number of visiting GPs is unrestricted. The role of pharmacists is mainly limited to the delivery of medications. Standardized medication reviews are not recommended or funded as in Australia or the United States (U.S.A.).

### Study design

We followed the Medical Research Council guidance on process evaluation of complex interventions [19]. The three domains of evaluation recommended by the guidance were explored, namely implementation, causal mechanisms, and contextual factors [19]. The “implementation” domain is the exploration of which elements of the intervention are actually delivered (dose), how delivery is achieved (fidelity and adaptations), and whether the intended target group comes into contact with the intervention (reach). The “mechanisms of impact” exploration aims to identify the process through which the intervention produces changes. The third domain involves identifying context elements that positively or negatively affect implementation and outcomes.

Data were collected using a mixed-methods approach combining quantitative and qualitative data. The data collection methods and main aspects evaluated are summarized in Table 2.

**Table 2:** Data collected for process evaluation

Aspects evaluated		Data collection method
1. Blended training: e-learning + interdisciplinary workshops		
Implementation	Who (which HCP) participated in which aspects of the training (e-learning course and workshops)	(Qt) Automatic recording of participation on the e-learning platform—attendance form for workshops
Mechanism of impact	Satisfaction (according to levels 1 and 2 of the Kirkpatrick model)	(Qt) Satisfaction survey
	Perceived effect on ICCs (Qt)	(Ql) Multidisciplinary focus groups
2. Local interdisciplinary meetings		
Implementation	Number of LIM sessions, number and types of participants, duration, level of consensus reached, etc.)	(Qt) Form filled in by the CP or (head) nurse after each LC
Mechanism of impact	Experiences and opinions of participants, satisfaction, perceived benefits (e.g., impact on ICCs, impact on the use of the therapeutic formulary, etc.)	(Ql) Multidisciplinary focus groups
Contextual factors	Factors influencing the implementation and the perceived impact of LIMs	(Ql) Multidisciplinary focus groups
3. Interdisciplinary case conferences		
Implementation	Number of ICCs, number and types of participants, duration, DRPs identified and discussed, etc.	(Qt) Electronic form filled in by the HCPs on the web application after each ICC
Mechanism of impact	Experiences and opinions of participants, satisfaction, perceived benefits (e.g., impact on medication use, on NHRs, etc.)	(Ql) Multidisciplinary focus groups
Contextual factors	Factors influencing the implementation and the perceived impact of ICCs Views on implementation on a larger scale in Belgium	(Ql) Multidisciplinary focus groups

*CP*: coordinating physician, *DRP*: drug-related problem, *HCPs*: healthcare professionals, *ICCs*: interdisciplinary case conferences, *LIMs*: local interdisciplinary meetings, *NHs*: nursing homes, *NHRs*: nursing home residents, *Ql*: qualitative data, *Qt*: quantitative data

### Quantitative data collection

Quantitative data were collected from the 24 intervention NHs.

For the training component, participation in an e-learning course was registered automatically through an e-learning platform and participation in the interdisciplinary workshops was registered using attendance forms. An online satisfaction survey was sent by email to healthcare professionals (HCPs) who took part in the training 3 months after the start of the training period. Two reminders were sent. In designing the survey, we focused on the first two levels of the Kirkpatrick model: reaction (to what degree participants react favorably to the training) and learning (to what degree participants acquire the intended knowledge, skills, attitudes, confidence, and commitment as a result of their participation) [28]. We formulated 25 statements and HCPs indicated their levels of agreement using a 4-point Likert scale (ranging from “strongly disagree” to “strongly agree”). The questionnaire was pilot-tested by three professionals with expertise in this area to check clarity, relevance, and completeness.

For LIMs, implementation data were collected through reports filled in by the coordinating physician and/or head nurse after each meeting and sent by email to the research team. Data collected included general information (topic discussed, number of participants, duration, etc.), level of consensus reached, and conclusions.

For ICCs, implementation data were collected during or straight after each ICC. The HCPs recorded the following information via a dedicated web application: participants, duration, preparation, drug-related problems identified, action taken, and follow-up.

### Qualitative data collection

Two months after the end of the study, qualitative data were collected using multidisciplinary focus groups (FGs) in a sample of intervention NHs. Purposive sampling was used to ensure variation in NH characteristics (size, ownership status, and location), the number of included NHRs per GP, previous experience with any form of ICCs, and the research team’s perception of implementation and success, based on regular contact between the research team and NHs. Twelve of the 24 intervention NHs were invited to participate. One contact person per NH was asked to invite eight to ten participants in total and at least one participant in each profession. One NH refused to participate due to lack of time.

Our aim was to collect information about how participants experienced the COME-ON study. In particular, the objectives were to understand in-depth how the intervention was implemented and how it produced changes and to identify barriers and facilitators affecting implementation and perceived impact. The results on the primary outcome were not available when the FGs met. Therefore, FG participants were invited to reflect on their perception of the determinants of the intervention’s success or failure. A semi-structured interview guide was developed and addressed each component of the intervention and each domain of the process evaluation. Participants were also asked to make recommendations for consideration if the intervention were to be scaled up across NHs in Belgium. The guide was tested during the first FG. Only minor modifications were made subsequently and, therefore, the first FG was retained in the final set for analysis. Each FG took place in a quiet room in the NH. One researcher moderated the discussion and a second was present as an observer and took field notes. The discussions were conducted in Dutch for the five NHs in Flanders and in French for the six NHs in Wallonia. Additional focus groups were not conducted because of the saturation of data. The mean duration was 106 minutes and on average eight HCPs were present. The coordinating physician, the pharmacist, and at least one nurse attended all FGs. For three FGs, no GP was present.

### Data analysis

Quantitative data were summarized using descriptive statistics using R software (Free Software Foundation, Inc., Boston, MA, U.S.A.).

All FGs were audio-recorded and transcribed verbatim in the original language. Participants were pseudo-anonymized by the assignment of an ID code. NVivo 10 was used to assist with the analysis. Researchers were experienced in qualitative analysis and their background (pharmacists and psychologist) differed so as to enable richer data interpretation.

A thematic analysis was performed using the pre-defined categories based on the themes in the interview guide. The first focus group was coded separately by three researchers (PA, MVG, SR; MVG and SR were external to the main trial team); MVG’s native language was Dutch but she had sufficient command of French to participate in the coding exercise for the first FG (held in French). Agreements were reached on the development of the coding tree after discussion among the three researchers and other members of the research team. Then, two researchers (MVG, SR) coded all transcripts for the focus groups held in their native languages. Consistency of coding was ensured through the use of a common coding book and through frequent meetings among researchers to discuss coding and, when needed, to edit the coding instructions. Afterward, the main barriers and facilitators that influenced the implementation and/or perceived impact of the intervention were classified using the four-level framework defined by Lau et al. [29]. The four key elements that influence the implementation of change include external contextual factors, organization-related factors, individual professionals, and characteristics of the intervention [29]. Researcher bias was minimized through regular cross-checking of data and findings by the members of the research team. We used quotations as exemplars of key themes.

### Ethical considerations

The study was approved by the Ethics Committee (s57145, ML11035) and by the Belgian Privacy Commission (SCSZ/14/084/174). All NHRs or residents’ representatives provided written informed consent. FG participants provided oral consent for audio-recording.

### Study registration

This study has been registered at http://www.isrctn.com/ (trial registration number: ISRCTN66138978).

## Results

### Blended training

In total, 71% (268/378) of all HCPs in the intervention group participated in the training. Participation rates varied according to profession, with GPs having the lowest participation rate. Full implementation data are presented in Table 3.

**Table 3:** Implementation data for each component of the COME-ON intervention

1. Blended training	
Participation rate in the blended training*, % (*n*)	71% (268/378)
Participation rate in the blended training^†^ per profession, % (*n*)	
Coordinating physicians	96% (23/24)
General practitioners	47% (90/192)
Pharmacists	87% (41/47)
Nurses	99% (114/115)
Participation rate in the e-learning course, % (*n*)	44% (166/378)
Participation rate in the interdisciplinary workshops, % (*n*)	61% (232/378)
Participation rate in both the e-learning course and the interdisciplinary workshops^†^, % (n)	34% (130/378)
Number of participating HCPs per NH	
Median Min–max	10 4–36
2. Local interdisciplinary meetings	
Number of LIMs, *n*	46
Proportion of NHs that organized two LIMs, % (*n*)	92% (22/24)
Number of HCPs per LIM per NH	
Median Min–max	12 6–28
Participation rate per profession, %	
Coordinating physicians General practitioners Pharmacists Nurses	100% 57% 61% 77%
Proportion of LIMs with all 3 professions represented, % (*n*)	83% (38/46)^‡^
Proportion of LIMs to which an expert was invited, % (*n*)	15% (7/46)
3. Interdisciplinary case conferences	
Number of ICCs per NHR	
Median Min–max	3 1–4
Proportion of NHRs with no ICC, % (*n*)	15% (123/804)^§^
Proportion of NHRs with at least 3 ICCs, % (*n*)	50% (403/804)
Proportion of NHRs with at least 3 ICCs among NHRs who completed the study, % (*n*)	70% (391/557)
Proportion of ICCs with all 3 HCPs present, % (*n*)	90% (1506/1675)
Time for preparation, median (min–max)	
General practitioners Missing data Pharmacists Missing data Nurses Missing data	10 (2–15) 62% (978/1580) 15 (3–100) 45% (690/1537) 15 (3–200) 73% (1160/1597)

*HCP* healthcare professional, ICC interdisciplinary case conference, LIM local interdisciplinary meetings, *NH* nursing home, *NHR* nursing home resident

* Participants who completed at least one e-learning module and/or attended the interdisciplinary workshop

† Participants who completed at least one e-learning module and attended the interdisciplinary workshop

‡ For 6 LIMs, the pharmacist was not present; for 4 LIMs, no GP was present. In 2 NHs, no GP attended either LIM

§ Most of these NHRs (*n* = 117) left the study, mainly because of death (*n* = 87)

One hundred and twenty-nine HCPs answered the satisfaction survey (response rate 48%, 129/268). Overall, the blended training was highly appreciated. With regard to the e-learning, 90% of respondents found that the content met their personal needs and 80% found its level of difficulty reasonable. Ninety-four percent self-reported an improvement in knowledge about appropriate medication use in older people and 98% in knowledge about conducting an interdisciplinary medication review. Ninety-four percent saw the content of the interdisciplinary workshops as relevant to COME-ON activities. In general, participants felt comfortable about discussing cases with colleagues, but fewer nurses (78%) and pharmacists (70%) than physicians (95%) felt comfortable about discussing the appropriate use of medicines with other professions. The interdisciplinary aspect was highly appreciated: 92% of respondents agreed that the workshop had stimulated collaboration with other HCPs within their NH. Furthermore, most respondents indicated an improvement in their confidence in actively participating in ICCs (84%) and in the skills required for performing a medication review (88%). The combination of the e-learning platform and the interdisciplinary workshops was considered “complementary” by 91% of respondents. Detailed results of the satisfaction survey are presented in Additional file 1.

Overall, data from the FGs confirmed the results of the satisfaction survey. Although the flexibility of the e-learning was appreciated by some HCPs, others—mainly nurses—found it difficult to find time to do it during working hours and showed reluctance to do it at home. The interdisciplinary workshop was preferred by some HCPs because of its interactivity and its planned/scheduled aspect, as well as by those lacking computer skills.

### Local interdisciplinary meetings

The majority of the NHs (22/24) carried out two LIMs. Two NHs organized only one meeting because of planning difficulties, even when the first meeting was appreciated, or unwillingness, because no benefit had been perceived after the first LIM. Participation rate per profession varied from 57% for GPs to 100% for coordinating physicians. An interdisciplinary team (i.e., at least one GP, one pharmacist, and one nurse) was present at most meetings (83%, 38/46). In two NHs, no GP attended either LIMs. In one of these, the coordinating physician did not dare to invite GPs because he considered that the workload due to the study was too high. On the other hand, he believed the notions discussed during the LIM should already be familiar to the other GP. Full implementation data are presented in Table 3.

A large majority of NHs reported having achieved a partial or complete consensus on the use of both medication classes for which support material was provided, i.e., antidepressants (91%, 21/23) and lipid-lowering drugs (95%, 20/21). However, data from the FGs show that opinions were divided on the level of consensus and the perceived impact of the LIM. Consequently, perception of the value of scaling up LIM meetings in Belgian NHs differed.

Some participants stated that the meetings did not have an impact because no real consensus was reached due to the lack of constructive discussion and/or the low level of GPs’ attendance. Even when a consensus was reached, some HCPs did not perceive an impact and/or were not sure that it led to changes in prescribing habits. The reasons cited included GPs were not convinced by the principle of selecting one or two first-choice drug(s), GPs clearly stated that they were attached to their therapeutic freedom, and the goal of the study was not always understood (e.g., fear of control). Other reasons included the feeling that no improvement was needed and the observation that the LIM was too late in the study to have an impact on the ICC. Participants in some NHs, however, perceived a positive impact: discussion of a specific medication class made it possible to bear the latest evidence in mind and made it easy to apply what they had agreed to the participating NHRs during the ICCs and also more broadly to all NHRs.

In spite of these mixed feelings on perceived impact, the LIMs seem to have been generally appreciated, especially the training aspect and the exchange of experiences. Nurses, in particular, but also some GPs, recognized that they learned something about the medication classes discussed. Moreover, with this extra knowledge, nurses reported feeling more confident about asking GPs questions about medications for individual NHRs.

The main barriers and facilitators that influenced the implementation and perceived impact of the LIM are presented in Table 4.

**Table 4:** Factors that influenced the implementation and/or the perceived impact of local interdisciplinary meetings

	Factors*	Implementation	Perceived impact	Quotes
Intervention	The interdisciplinary approach: requirement to gather a maximum of HCPs for the LIM [Complexity of implementation process]	Barrier		GP-F2: “The most difficult part of the local interdisciplinary meeting, perhaps, was finding a suitable moment for it. Because a larger number of physicians had to be present. I think that was the biggest problem, to arrange the agenda so everyone could attend.”
	The overall workload of the COME-ON intervention [Nature and characteristics]	Barrier		CP-F3: “But I didn’t invite any GPs [since the workload was already so high for GPs in the COME-ON study]. It was the last step that I didn’t dare to take.”
	Class of medication to be discussed [Nature and characteristics]		Facilitator	PH-W4: “It’s much easier to arrive at an idea, at a consensus with lipid-lowering drugs than with antidepressants, for many different reasons. In particular, because we have very objective criteria in the case of lipid-lowering drugs.”
			Barrier	CP-F5: “There are a number of parameters for lipid-lowering drugs. But if you start to discuss the topic of antidepressants, there are so many factors involved that are not only pharmacological. When the parameters are much more difficult, it’s a bit guesswork for depression, it’s not so easy to distinguish.”
	Material (i.e., summary of the evidence + topics to be debated) provided by the research team [Nature and characteristics]	Facilitator		GP-W2: “The PowerPoint presentation [a document provided by the research team with a summary of the key aspects and points to be discussed] was very helpful.”
			Facilitator	GP-F2: “I think that, with regard to the physicians, it is important that all those learning modules are available to refresh their knowledge. Especially from the discussions between the GPs at the beginning, for example, the first one was about statin use for those aged 65 and over. At the beginning of the conversation, there were ten different approaches, but we were still able to achieve a consensus at the end. If you did it without any preparation, I’m not sure whether drugs would be tapered so smoothly.”
	Expert invited to contribute to the discussion [Nature and characteristics]		Facilitator	CP-W2: ”I think that Dr X [a geriatrician who was the invited expert], who came to the first local interdisciplinary meeting shared his experiences and encouraged people to think about things. /…/ It was very helpful.”
Professional	Perceived relevance or utility of LIM from a GPs’ point of view [Attitudes to change]	Facilitator		DIR-F2: “I also think we have a highly motivated group. I don’t remember whether it was the first or the second local interdisciplinary meeting, but there were GPs who said when they were leaving, ‘we should really do this again for other medication classes.’ That was a comment from a GP as he left after the local interdisciplinary meeting consultation.”
		Barrier		PH-W6: “So [lack of perceived interest on the part of the GPs] we didn’t organize a second local interdisciplinary meeting. You had asked us [in accordance with the study protocol] to organize two local interdisciplinary meeting, but we didn’t feel they were interested in those meetings.”
			Barrier	GP-W6: “In my opinion, the discussions were too theoretical and, well … Sometimes, it seems to me that it’s just empty talk.”
Organization	Implementation of decisions taken during LIM [Process and system]		Facilitator	MCC-W2 : “What worked well was that, when we reached a consensus during the LIM, we knew the impact that it could have on the next ICC, on the follow-up of our patients.”
			Barrier	HN-W1: “ /…/ It’s true, that’s what we decided [during the LIM about the appropriate use of antidepressants], we’re going to do it, we’re not doing it! /…/ But we had already forgotten, to some extent...”
	Local aspect—between HCPs who already know each other [Relationship]		Facilitator	CP-W2: “The fact that it was local, people knew each other already, and everybody was taking part in the study, so we were all in the same boat…it wouldn’t have the same impact if the meetings [local interdisciplinary meetings] had been organized in Brussels with all the nursing homes /…/ in one big gathering.”
External context	Influence and expectations of health authorities [Policy]		Barrier	CP-W4: “We did feel that it was only about medications that are reimbursed. In my opinion, benzodiazepines should have been included too and not just antidepressants. We felt the INAMI [National Institute for Health and Disability Insurance] was behind that.”
	Funding	Facilitator		GP-F2: “On a systemic basis, I think it could cause some problems without any remuneration.”

*CP*: coordinating physician, *DIR*: directory board, *F*: Flanders, *GP*: general practitioner, *HCPs*: healthcare professionals, *HN*: head nurse, *LIM*: local interdisciplinary meeting, *PH*: pharmacist, *W*: Wallonia

* According to the framework defined by Lau et al. [27]

### Interdisciplinary case conferences

A total of 1675 ICCs were registered for 681 NHRs. The median number of ICCs per NHR was 3 (P_25_-P_75_, 2–3; range, 1–4). The proportion of NHRs with at least three ICCs varied greatly between NHs, from 0% to 100% (median 84%, [P_25_-P_75_] 54-94). Full implementation data are presented in Tables 3 and Additional file 2.

For 90% of ICCs, at least one nurse, one pharmacist, and one GP were present. The median duration of ICC meetings was 15 minutes.

The median number of drug-related problems (DRPs) recorded per NHR was 4.8, but varied from 0 to 8 in different NHs. The median number of DRPs per NHR was 2 for the first ICC and 1 for the second and third ICCs (Additional file 2). FG participants saw the first ICC as the most important, as many DRPs were discussed there. The second and the third ICC were seen as useful follow-ups by some HCPs and as unnecessary by others.

Overall, participants were satisfied with the ICCs. They appreciated time being taken to have an interdisciplinary patient-centered discussion about medications, under better conditions than the short exchanges that occur during routine GP visits. The study raised their awareness of the need to conduct regular medication reviews. However, the perceived impact of the ICCs on NHRs varied greatly. Some HCPs mentioned that few treatment changes were implemented, either because they considered them unnecessary or due to inertia or reluctance to change. Moreover, they stated that it was more difficult to change a medication prescribed by a specialist physician or medications for NHRs with psychiatric disorders. Some HCPs were unable to identify whether or not the ICC had an impact. Others reported an impact in a reduction of the number of medications and sometimes a cost reduction. Improvements in NHRs’ conditions and/or quality of life were seldom reported.

Besides perceptions of the impact on NHRs, participating HCPs recognized some benefits for themselves. Some nurses reported being more confident about asking GPs questions about medications and being increasingly involved in the resident’s follow-up. Some pharmacists stated that, thanks to the study, other HCPs started to acknowledge their role. Moreover, the relationships between the HCPs involved improved throughout the study.

Globally, residents and relatives were not informed or only briefly informed about decisions taken at ICCs. The presence of NHRs would have been perceived as a barrier; although most participants mentioned that it is important to involve residents and/or relatives to some extent, they did not know how exactly this should be done.

The main barriers and facilitators affecting the implementation and impact of ICCs are presented in Table 5.

**Table 5:** Factors that influenced the implementation and/or the perceived impact of the interdisciplinary case conferences (ICCs)

	Factors*	Implementation	Perceived impact	Quotes
Intervention	Face-to-face approach [Nature and characteristics + implementability]	Barrier		PH-W1: “On the other hand, in relation to timing and planning, it wasn’t easy…/…/ we met several general practitioners, one after another, we didn’t know how long that would take. So sometimes we had to wait half an hour or an hour and sometimes we hadn’t finished and the GP had to wait a quarter of an hour. So, timing wasn’t easy… /…/ because we all have our own very busy schedules.”
			Facilitator	PH-W2: “The fact that we took the time, we were all around the table, it was much more convivial too and there was real sharing… Just sending e-mails is less effective.”
	Interdisciplinary approach with three different HCPs [Nature and characteristics + implementability]	Barrier		HN-W1: “[About the organization] It is necessary to be quite conscious that to gather everyone around the table, it’s a complex balancing act. And that it’s not always easy.”
			Facilitator	CP-F1: “I found it worked well with those three [GP, pharmacist, and nurse]. You shouldn’t do it with fewer – then you’re lacking one of the keys.”
	Preparation of the ICCs [Nature and characteristics]		Facilitator	CP-W2: “/…/ I think one secret [for an effective interdisciplinary case conference] was to prepare the meeting properly. I think that when she [the pharmacist] came, she had done her homework in a way I hadn’t. So that was very helpful.”
	Material (i.e., summary of the evidence) provided by the research team [Nature and characteristics]		Facilitator	PH-W2: “I worked a lot with the summary sheets [the research team provided some summary sheets about various topics: e.g., a short list of STOPP-START criteria, a list of medications with anticholinergic activity, etc.], which were quite well done. I shared those with my colleagues, because they are very interesting tools on which I relied during the discussion.”
Professionals	GPs’ motivation to participate [Attitudes to change]	Facilitator		HN-W1: [About identification of facilitators for success] “We have about 90 generalists who attend our institution and I actually chose them [GPs who participated in the COME-ON study].../…/ I think, in terms of the choice in the first place and the motivation, they have already agreed to be part of the project ...halfway convinced.”
		Barrier		HN-W2: “I mean that the GPs who were not interested or not motivated, well, they refused to participate. We’ve had quite a few refusals to participate.”
	Interprofessional relationships and clarity of role and responsibility [Professional role]		Facilitator	GP-F2: “I think that, because you have sat around the table with each other more – and that always works in my opinion – you feel more part of a team. If you go to a NH for the first time and you don’t know anyone there. But if you have been able to discuss things with those people a few times, then you know who you are dealing with and who you are working with. But that’s not only due to this project. There are many things that contribute. If you collaborate in relation to a very difficult resident or a very difficult situation, then you also learn to work together and you get to know the people you work with a bit.”
			Barrier	CP-W3 : “But I think in your job as a pharmacist, you have to be aware of ‘what doesn’t go with what [drug-drug interactions]’… /…/ I think it is necessary for everyone to bring his/her expertise but everyone must still be in his/her own job. So the physicians choose the treatments according to the indications...and the pharmacist...makes sure that everything is in...that’s how I see it! It doesn’t bother me that she [the pharmacist] has access to diagnoses. That’s not the problem but...but I think that if she [the pharmacist] wants to have an expert opinion on medications, it is not the diagnoses that are going to help her.”
	GPs open to suggestions from other HCPs		Facilitator	GP W1 :“I think that the atmosphere was positive. In the discussion ([ICC], the GP did not insist on always being right; so there was a real collaboration, with all three (GP, pharmacist, and nurse] working together with a shared goal.”
			Barrier	DIR-F5: “And I think that, nothing to do with the individuals as such, but it has to do with, how should I say this, the overall perception of physicians and pharmacists. That there still is a bit of a tension between physicians and pharmacists… It has to do with the therapeutic freedom of physicians, who still participate all the time but don’t really like it when a pharmacist interferes with prescribing. Although I think the input from the pharmacist is really great, I think physicians don’t like it.”
	Lack of skills, knowledge, and experience of pharmacists to conduct a medication review [Competency]		Barrier	PH-W1: “It was new. And besides asking [basic] questions [on medication regimens] like ‘Why is it like that?’ […]...unlike the clinical pharmacist, it's still a minimum of three more years of study, after all, it's different...to start discussing things with the physicians...so, between theory and practice..."
Organization	Those of the nursing staff not involved in ICCs [Involvement]		Barrier	DIR-F5: “At a certain moment, we sat down together here and we said we are operating on two different wavelengths. One the one hand, we have the COME-ON team that goes on step by step. And we haven’t done enough to involve the people in charge here, who are involved in the daily care for these people and who therefore also see possible side effects of medication and who should actually implement what has been discussed at the ICC. They haven’t had enough opportunities, and it’s all our fault, to thoroughly apply all the information that was available.”
	Lack of nursing staff resources [Resources]	Barrier		HN-W4: “It was really [difficult/complicated] to release someone [a nurse], at times that, moreover, were difficult here in the nursing home, when there was a pretty well record rate of absenteeism. And it was hard for me to tell my management. Well, I'm taking four nurses .../…/ to spend half a day reviewing the treatments. It's really complicated.”
	Availability of the delivering pharmacist [Resources]	Barrier		HN-W1: “In contrast, it was X [pharmacist] and her timetable that got hit, rather than the general practitioners.”
	Previous experience of interdisciplinary collaboration/pre-existing relationship between HCPs [Relationship]	Facilitator		CP-W3: “…and so we had a team that knew each other well, who already met often...and I wonder about the implementation in practice of something like that with the other general practitioners come to the nursing home.”
	One person responsible for the planning, who motivated the team [involvement]	Facilitator		HN-F2: “Dr XX (coordinating physician of the NH) first asked which days suited them best. We took that into account. But then we drew up a schedule for the year, with all the ICCs planned from the start. And yes, send a reminder a week beforehand, before we started the next series, and sometimes a reminder to the physicians two days in advance as well. So that’s everything sorted out [laughs]. But I do understand that it’s important.”
	Logistical resources: e.g., a quiet room, access to the resident’s records and medication schedule during ICC, computer, Wi-Fi connection [Resources]		Facilitator	CP-W2: “…and at a practical level, but which was in the specifications [required in the protocol], is to have a specific room with a computer and a Wi-Fi connection. That was really very useful, because if it had to be done in the nurses’ station while other nurses are preparing drugs or when you have patients coming in all the time and so on, it would have been... So having the adequate infrastructure was necessary.”
External context	Financial incentive	Facilitator		PH-F4: “The payment: for sure, in a study context, that’s nice to have. I think for implementation on a wider scale, now we’re only speaking of 30 patients, but there will be more patients, it will be more frequent, there will be more work to do too, and there has to be something in return, otherwise it’s not feasible.”
	Clinical trial context	Facilitator		CP-W1: “We’re doing it this time because this time we're in a... [study]; we've accepted a contract and we respect it.”

*CP*: coordinating physician, *DIR*: directory board, *F*: Flanders, *GP*: general practitioner, *HCPs*: healthcare professionals, *HN*: head nurse, *ICCs* interdisciplinary case conferences, *PH*: pharmacist, *W*: Wallonia

* According to the framework defined by Lau et al. [27]

First, the main intervention-related facilitators were the interdisciplinary and face-to-face approaches, which contributed to the success of ICCs, despite the organizational constraints. Likewise, the preparation made, mainly by pharmacists, was considered an advantage. Second, at the level of professionals, we found variability in expectations regarding the roles of other HCPs. This was particularly true of the role of the pharmacist, whose contribution some found positive and others confronting. A positive attitude by GPs was acknowledged as a facilitator when present and its lack was seen as a barrier. Third, the key facilitator at the organizational level was the presence of a leader who promoted/stimulated the implementation of the intervention. Preexisting positive relations between the HCPs involved also facilitated the intervention. Finally, HCPs were paid for their participation in the COME-ON study. This external contextual factor was appreciated and could be seen as a facilitator.

For future implementation, FG participants raised concerns about feasibility, mainly due to time and resource constraints and a lack of motivation among non-participating GPs. Funding would have to be provided and they suggested reducing the number of ICCs per resident from three to one or maximum two per year and stated that hospital discharge could offer a good opportunity to perform a medication review, even if this had not been performed during the study.

### Discussion

This process evaluation used a mixed-methods approach in order to understand to what extent the COME-ON intervention was implemented as foreseen and to explore participants’ experiences. It made it possible to identify barriers and facilitators, to reflect on how implementation may have influenced outcomes and to highlight lessons for future implementation. Overall, implementation and satisfaction were good, which probably contributed to the positive effect seen on the primary outcome measure, but variation was observed between NHs and HCPs. Several elements might explain the variability in implementation and perceived impact. These are discussed below.

### Intervention

The training component was highly appreciated—including its interprofessional aspect—and was perceived as an important step before LIM and ICCs. The online training and the face-to-face workshops were considered complementary and we believe that both approaches should be offered in the future. Even though there is little current evidence of the impact of educational approaches on the quality of prescribing [14, 18], it is often considered an essential component of complex interventions. Moreover, in a recent study, GPs’ knowledge of geriatric pharmacotherapy improved after a short e-learning module [30]. It is also noteworthy to comment that the medication classes on which an effect was seen in the intervention group (e.g., proton pump inhibitors, vitamin D) were all included in the “short list of frequent PIMs and PPOs”. This list was a core component of the training provided. Several participants of the focus groups highlighted the usefulness of this list, which is reflected in these data. The innovative aspects of the COME-ON training were the combination of e-learning and onsite training and its interprofessional approach. As far as we know, this approach had never been tested in the nursing home setting.

Opinions on LIMs diverged. While the training aspect and the exchange of experiences were generally appreciated, divergent views were expressed on the impact on prescribing and NHRs. Many GPs did not fully support the aim of reaching consensus on the drugs to be integrated into a therapeutic formulary, as it was perceived as limiting physicians’ freedom in prescribing. An evolution in attitudes on this matter requires time. The focus group data suggest that the class of medication discussed during LIMs may influence impact, i.e., that for more complex themes (antidepressant was mentioned as more complex that lipid-lowering drugs), the implementation may be lower. This is in line with the findings on medication use data, which show a significant decrease in the use of lipid-lowering drugs, but no major modification in the use of antidepressants. Given the positive experiences reported with LIMs in other countries such as Switzerland [31], the low cost, and complementarity with ICCs, we believe that LIMs—with clear instructions and adequate material—should be maintained and be scaled up in the future. The interprofessional aspect of both training and LIM is key, as our data suggest that this enabled the development of a common language across the team and improved communication processes. This may have a downstream effect on the appropriateness of prescribing [24].

The interdisciplinary and face-to-face approaches of ICCs were identified as facilitators for the success of the intervention, but also as barriers to implementation due to organizational constraints. The face-to-face approach has been acknowledged to contribute to effective teamwork because it fosters the establishment of a trusting relationship [32, 33]. Therefore, for future implementation, it seems important that ICCs are conducted face-to-face. However, as suggested during the FGs, once a trusting relationship has been established, other forms of communication (e.g., teleconsultation) would be potential alternatives. Overall, participants saw the workload and frequency of ICCs as heavy and most were concerned about the feasibility of scaling up ICCs to all NHRs. As in other countries [34], a yearly interdisciplinary medication review for each NHR seems more appropriate.

### Professional

Consistently with the literature, the presence of the GP during ICCs was regarded as essential [35]. However, GP participation in the different components of the intervention was a challenge, even though the participant GPs were volunteers. Several factors were identified: organizational constraints, lack of motivation, the goal of the study is unclear, unclear expectations, reluctance to have their therapeutic decisions questioned, and a large number of GPs per NH. These factors have already been mentioned in the literature [36]. In many countries where an open staff model composed of independent physicians is the norm, there is a need for more medical governance in long-term care, in order to improve the accountability of attending physicians [37]. Our results confirm that the role of the coordinating physicians should be strengthened and that NHs must develop a stronger policy on medication use that must be accepted by all attending GPs.

In contrast to studies in other countries such as the U.S.A. and Australia [38], actively involving pharmacists was an innovative step. We observed variations in GPs’ expectations of the role of pharmacists. Given the lack of close collaboration in daily practice with community pharmacists, GPs might not acknowledge their expertise [32]. A clear definition of roles and responsibilities with regard to medication review is required, as it is one of the key determinants of effective interprofessional collaboration [33, 39]. Another common barrier to effective collaboration with GPs is inadequate clinical training of pharmacists [40]. In the present study, some pharmacists stressed their lack of knowledge and skills in medication review as an important barrier. Consistently with the literature, we strongly believe that all HCPs involved could benefit from additional training in geriatric pharmacotherapy [41]. However, if there is a willingness to extend the pharmacists’ role in the NH setting, they certainly need more training. Taking a lead from Australia, we could imagine mandatory training for pharmacists to obtain accreditation for working in the NH setting [34].

### Organization

There were also factors in NHs as organizations that had a positive influence on the implementation and impact of the intervention, such as preexisting relations between HCPs [42], the presence of a leader, and staff resources. The organizational culture and prescribing culture may also have influenced the implementation and impact [43, 44]. Several studies have suggested that the culture in NHs is linked to variations in the use, for example, of psychotropic medicines [45, 46].

### External context

Funding was provided for ICCs and certainly contributed to the good implementation rates. It seems obvious that future implementation of ICCs must be remunerated. The clinical trial context also positively influenced implementation. Particular attention should be paid to real-world implementation data, if any component of our intervention is to be adopted in routine practice.

## Strengths and limitations of this study

This study is one of the first studies to have conducted an in-depth pre-planned process evaluation of an intervention implemented in NHs that aimed to improve the quality of prescribing [24, 19]. A number of important findings emerged from the process data; these help us to understand the implementation of the intervention and participant satisfaction and to identify lessons for future implementation. Examples of final recommendations made to Belgian stakeholders at the end of the study and for which process data strongly influenced the recommendation are provided in Additional file 3.

However, this study has some limitations. We used FGs to collect qualitative data. Participatory observation supplemented by individual interviews could have provided an even fuller picture of how different factors influenced implementation and impact, but this was not possible due to time constraints. Due to the multidisciplinary nature of FGs, participants may have been reluctant to mention barriers relative to interprofessional collaboration in front of other HCPs. The FGs were moderated by members of the COME-ON study team, some of whom had regular contact with participating NHs, so some participants may have felt uncomfortable about voicing their views. Moreover, FG participants may also have been more enthusiastic HCPs. However, we observed variations in opinions and experiences between FGs. Therefore, we can reasonably assume that the process evaluation made it possible to identify the main enablers and barriers. We did not explore the views of NHRs or relatives. Finally, we did not quantify associations between the present process data (e.g., level of implementation, HCP or organization-related factors) and the primary outcome measure. Those should be studied in future research.

## Conclusions

The process evaluation of the COME-ON study shows that the intervention was, overall, well implemented, including with regard to its interprofessional aspect, and was appreciated by HCPs under certain conditions. Careful development and piloting of the intervention likely contributed to good implementation and satisfaction data, and these in turn probably contributed to the positive effect seen on the appropriateness of prescribing. The current process evaluation data also suggest that a larger effect could be reached with several adaptations such as reinforcing the role and competency of the pharmacist, strengthening interprofessional relationships, and improving medical governance and GP’s accountability. Future implementation must take into account the various factors—at the intervention, professional, organizational, and external context levels—that were found to affect implementation or impact, in order to maximize effect and sustainability.

Contributions to the literature

- Studies of complex interventions to improve appropriateness of prescribing in nursing homes have produced mixed results.
- In this controlled trial with a favorable effect on appropriateness of prescribing, implementation of the intervention was generally good. We identified elements at various levels—the intervention, professionals, organization, and external context—that may explain variations in implementation and impact. These elements are consistent with or complement the existing literature.
- These findings contribute to recognized gaps in the literature on the optimization of prescribing in nursing homes, including describing the level of implementation, identifying mechanisms of impact and contextual influences.

## Supplementary information

**Additional file 1.** Blended training: satisfaction survey - Results.

**Additional file 2.** Quantitative data on the implementation of ICCs.

**Additional file 3.** Examples of final recommendations made to Belgian stakeholders, derived from the process evaluation data.

## Data Availability

Part of the data generated or analyzed during this study is included in this article and its supplementary information files or is available from the corresponding author on reasonable request. The full datasets generated or analyzed during the current study are not publically available because consent to make data publically available was not part of the consent by participants.

## Abbreviations

COME-ON: Collaborative approach to Optimize Medication use for Older people in Nursing homes
FG: Focus group
GP: General practitioner
HCP: Healthcare professional
ICC: Interdisciplinary case conference
LIM: Local interdisciplinary meetings
NH: Nursing home
NHR: Nursing home resident
PIP: Potentially inappropriate prescribing
RCT: Randomized controlled trial
USA: United States of America
