# Supplementary material for: Process evaluation of a complex intervention to optimize quality of prescribing in nursing homes (COME-ON study)
Source: Implement Sci. 2019 Dec 11;14:104. doi: 10.1186/s13012-019-0945-8 (PMC6907338; doi:10.1186/s13012-019-0945-8)
Supplement: Supplementary file 1 — Additional file 1. Blended training: satisfaction survey - Results. [file 13012_2019_945_MOESM1_ESM.docx]

| **Additional file 1.** Blended training: satisfaction survey - Results | | | | |
| --- | --- | --- | --- | --- |
|  | Level of agreement, % (n) | | | |
|  | Strongly disagree | Disagree | Agree | Strongly agree |
| Part 1. E-learning platform | | | | |
| Level 1: Reaction | | | | |
| Time taken to complete the course | | | | |
| Time required to complete one module (mandatory parts for accreditation) on the e-learning platform is too long | 3% (4) | 44% (50) | 44% (50) | 9% (10) |
| Online navigation | | | | |
| The e-learning platform is easy to use (login procedure, navigation, playing video,…) | 0 % (0) | 11% (14) | 66% (75) | 23% (26) |
| Course presentation | | | | |
| The way the content is presented raises my interest to discover it. | 1% (1) | 14% (16) | 73% (83) | 12% (14) |
| Course relevance | | | | |
| The content provided on the e-learning platform meets my personal needs for the COME-ON Study | 0% (0) | 10% (11) | 81% (92) | 10% (11) |
| Global satisfaction | | | | |
| The content of this e-learning platform was reasonable in difficulty for me with my background | 1% (1) | 19% (22) | 72% (82) | 8% (9) |
| I would recommend other HCPs of my own profession to use this Come-On e-learning platform | 0% (0) | 14% (16) | 68% (77) | 18% (21) |
| Level 2: Learning | | | | |
| Knowledge | | | | |
| After going through this e-learning platform, my knowledge has improved about appropriate medication use in older people | 0% | 6% (7) | 56% (64) | 38% (43) |
| After going through this e-learning platform, my knowledge has improved about how to perform an interdisciplinary medication review | 0% | 2% (3) | 73% (83) | 25% (28) |
| Skills | | | | |
| After going through this e-learning platform, my skills in contributing a medication review have been improved | 1% (1) | 3% (4) | 73% (83) | 23% (26) |
| Confidence | | | | |
| After going through this e-learning platform, I’ve become more confident to actively participate to interdisciplinary case conferences. | 0% | 18% (20) | 62% (71) | 20% (23) |
| Commitment | | | | |
| After going through this e-learning platform, I will start using what I learned outside of the COME-ON study | 1% (1) | 1% (1) | 70% (80) | 28% (32) |
| General question | | | | |
| I appreciated learning through this particular e-learning platform | 0% | 7% (8) | 71% (81) | 22% (25) |

| Additional file 1. Blended training: satisfaction survey – Results (continued) | | | | |
| --- | --- | --- | --- | --- |
|  | Level of agreement, % (n) | | | |
|  | Strongly disagree | Disagree | Agree | Strongly agree |
| Part 2. Interdisciplinary workshops | | | | |
| Level 1: Reaction | | | | |
| Time taken to complete the course | | | | |
| The length of the face-to-face training was too long | 11% (11) | 76% (80) | 13% (14) | 0% |
| More sessions are needed to be ready to perform the activities of the COME-ON study | 3% (3) | 45% (47) | 48% (51) | 4% (4) |
| Course relevance | | | | |
| The content of this face-to-face training was relevant for the activities to be realized during the COME-ON study | 1% (1) | 5% (5) | 81% (85) | 13% (14) |
| Global satisfaction | | | | |
| During the face-to-face training, I felt comfortable to discuss about appropriate use of medication with other HCPs. | 0 % | 17 % (18) | 70 % (74) | 13 % (13) |
| Nurses (N=46) | 0%(0) | 22%(10) | 78%(36) | 0% |
| Coordinating physician (N=16) | 0%(0) | 6%(1) | 75%(12) | 19%(3) |
| GPs (N=23) | 0%(0) | 4%(1) | 57%(13) | 39%(9) |
| Pharmacists (N=20) | 0%(0) | 30%(6) | 65%(13) | 5%(1) |
| During the face-to-face training, there were good interactions with other HCPs of my nursing home. | 0 % | 3 % (3) | 68 % (71) | 29 % (31) |
| Generally speaking, face-to-face interactive meetings is a way of teaching that I appreciate | 0 % | 9 % (9) | 88 % (93) | 3 % (3) |
| Level 2: Learning | | | | |
| Confidence | | | | |
| The face-to-face workshop provided me with more confidence to actively participate in interdisciplinary case conferences. | 0 % | 16 % (17) | 78 % (82) | 6 % (6) |
| Skills | | | | |
| After the face-to-face workshop, my skills in performing a medication review have improved | 0 % | 12 % (13) | 84% (88) | 4% (4) |
| Attitude | | | | |
| I felt this interdisciplinary workshop has stimulated collaboration with other HCPs within the nursing home. | 0 % | 8 % (9) | 65 % (68) | 27% (28) |
| General question | | | | |
| I appreciated to attend this particular face-to-face session | 0 % | 4 % (4) | 78 % (82) | 18 % (19) |
| Part 3. E-learning + interdisciplinary workshops | | | | |
| The face-to-face workshop and the e-learning platform are both well complementary | 0 % (0) | 9 % (9) | 80 % (75) | 11 % (10) |
