# Supplementary material for: Process evaluation of a complex intervention to optimize quality of prescribing in nursing homes (COME-ON study)
Source: Implement Sci. 2019 Dec 11;14:104. doi: 10.1186/s13012-019-0945-8 (PMC6907338; doi:10.1186/s13012-019-0945-8)
Supplement: Supplementary file 2 — Additional file 2. Quantitative data on the implementation of ICCs. [file 13012_2019_945_MOESM2_ESM.docx]

| **Additional file 2.** Quantitative data on the implementation of ICCs | | | | |
| --- | --- | --- | --- | --- |
|  | 1^st^ ICC | 2^nd^ ICC | 3^rd^ ICC | 4^th^ ICC |
| Proportion of NHRs who were discussed, % (n) | 84.7% (681/804) | 72.4% (582/804) | 50.1% (403/804) | 1.1% (9/804) |
| Type of ICC, n  Planned  After hospitalization  Palliative care  *Missing data* | 627  -  -  *54* | 512  1  -  *69* | 379  -  -  *24* | 9  -  -  - |
| Duration, min *  Median [P_25_–P_75_]  Min–Max  *Missing data* | 15 [15–20]  5–120  *54* | 15 [10–20]  2–35  *69* | 15 [10–15]  1–35  *25* | 5 [5–5]  5–15  *0* |
| DRPs recorded | | | | |
| Number of DRPs recorded per NHR  Median [P_25_–P_75_]  Min–Max | 2 [1–4]  0–15 | 1 [1–2]  0–12 | 1 [0–2]  0–13 | 0 [0–1]  0–3 |
| Total number of DRPs recorded, n (%) | 1910 (56.4%, 1910/3386 ) | 912 (26.9%, 912/3386) | 561 (16.6%, 561/3386) | 3 (0.1%, 3/3386) |
| Proportion of ICCs with no DRP recorded, % (n) | 9.8% (67/681) | 22.9% (133/582) | 30.5% (123/403) | 66.7% (6/9) |

One NH was excluded because only one ICC was registered for the 43 NHRs included. Based on the data from the focus group, this does not reflect the reality.

GP: General Practitioner, ICC: Interdisciplinary Case Conference, NHR: Nursing Home Resident

*Additional time was required to complete data in the web application before and after the ICC, to prepare the ICC, to inform the nursing staff, the resident, and his/her family, and to edit the medication schedule. Time spent on preparation by each HCP was measured. General practitioners spent a median of 10 minutes on preparation [10-15], pharmacists 15 minutes [10-20], and nurses 15 minutes [10-15]). However, around 50% of preparation times were missing and we did not collect data on the nature of preparation.
