# Supplementary material for: Process evaluation of a complex intervention to optimize quality of prescribing in nursing homes (COME-ON study)
Source: Implement Sci. 2019 Dec 11;14:104. doi: 10.1186/s13012-019-0945-8 (PMC6907338; doi:10.1186/s13012-019-0945-8)
Supplement: Supplementary file 3 — Additional file 3. Examples of final recommendations made to Belgian stakeholders, derived from the process evaluation data. [file 13012_2019_945_MOESM3_ESM.docx]

**Additional file 3**: Examples of final recommendations made to Belgian stakeholders, derived from the process evaluation data.

The final set of recommendations was directed towards deciders, nursing homes (NHs) and health care professionals (HCPs). They were mainly based on the results from the Come-On study, but also on international data. Ongoing reforms and evolutions in the organisation of (primary) health care in Belgium were also taken into account. The recommendations result from a three stages Delphi process involving the research team, steering committee and experts.

The subset of recommendations listed below represents examples of recommendations (partly) derived from the process evaluation data. The detailed set of recommendations (including detailed recommendations and rationale) is available from the authors upon request.

A.Recommendations on Education and training

Recommendation A2: An advanced level course on appropriate medication use in older people and on the importance and process of medication review must be part of continuing education / continuous professional development for nurses, pharmacists, GPs and possibly other HCPs who take up a role in the care of older people.

Recommendation A3: To enhance interdisciplinary collaboration around the medication review process, inter-professional training is required, by which GPs, pharmacists, nurses and other HCPs can learn about, with and from each other.

B. Recommendations on local interdiscplinary meetings

Recommendation B1: Local interdiscplinary meetings (on the level of the NH) on the appropriate use of specific medication classes and alternative non-pharmacological approaches in older people must be organised at least once a year.

Recommendation B2: Material including the latest evidence, non-pharmacological approaches, benchmark information and relevant questions should be developed to support the local interdiscplinary meetings. Such material must be developed at the national level.

C. Recommendations on interdisciplinary case conferences (ICC)

Recommendation C3: A first ICC on medication review has to be organised within the first three months after the resident’s admission to the NH, and thereafter at least once a year. Additionally, ICC has also to be organised after each key transition moment (e.g. after hospitalization, when entering palliative care...).

Recommendation C4: The core interdisciplinary team of an ICC on medication has to include at least the resident’s GP, a pharmacist and a (chief) nurse involved in the care of the resident.

Recommendation C6: At least the first ICC for a new resident has to be organized face-to-face, in order to allow a deep and complete medication review taking into account the objectives for the resident, and to create a trust-based relation between the participants.

E. Recommendations on HCP

Recommendation E1: All HCPs involved in the care of older people in a specific NH, and particularly GPs, must be actively informed about the NH policy on rational drug use* (for instance: organisation of local interdiscplinary meetings, prescribing based on therapeutic formularies, ICC, contribution of and communication with the resident,…) and need to formally agree on that.

Recommendation E2: The (new) role of the pharmacist, as expert in medication, in the interdisciplinary team in the NH should be strengthened and developed towards a position as ‘Consultant Pharmacist’

Recommendation E3: The role of the coordinating physician in the NH should be strengthened in order to facilitate the development of a NH-specific policy on rational prescribing, the organisation of local interdiscplinary meetings and ICC, and the development and implementation of a therapeutic formulary

Recommendation E4: A concrete remuneration system for all HCPs participating to ICC on medication must be developed and implemented by political authorities and NHIDI, in collaboration with health insurers and professional organisations

F. Research agenda

Recommendation F3: As the results from the Come-On study about local interdiscplinary meetings are contrasting, it should be useful to further re-evaluate this component of the programme, in order to better identify the success factors and bottlenecks.

G. Implementation strategy

Recommendation G3: In order to support the implementation of the recommendations previously formulated on the rational use of medication, the creation of a "Support Cell" is required. The role of this ‘Cell’ should be the facilitation of the implementation of these recommendations with, for instance, the development of educational material, mobile support to local teams, the facilitation of exchange of experiences of local teams, …
